# 

**Published:** 1921-06-18

**Authors:** 


June 18 1921] [Price Twopence

### The Hospital

The Workers' Newspaper of Administrative Medicine and Institutional Life, Administration, National Insurance and Health.

### CONTENTS

**The Voluntary Hospitals Committee** ... ... 189

**Co-operation Schemes** ... ... ... ... ... 190

**Notes on the Cave Committee's Report** ... ... 190

**Notes of the Week** ... ... ... ... .. 191

A Human Document—The Children and Hospital Sunday—The Closed Wards—Rejuvenescence—Oil Fuel and Coal Difficulties—Full Huts and Empty Wards—Subscriptions from Local Authorities—Venereal Disease amongst Seamen—Opponents of Hospital Saturday—An Effect of Smoking in the Wards.

**On Comparative Medicine** ... ... ... ... 193

An Age of Specialism.

**The Prince at Bristol and Cardiff** ... ... ... 193

**Hospitals in Literature** ... ... ... ... ... 194

II.—" The Surgeon's Daughter," by Sir Walter Scott.

**Hospital Engineering Reforms** ... ... ... 195

III.—Electrical Thermometers and Boiler Temperatures.

**Pensions and Neurasthenia** .. ... ... ... 196

Some Reflections by an Ex-Patient.

**The Public Well-Being** ... ... ... ... ... 197

Sanitary Reform—A Lesson from America.

**Cholera at Salonica** ... ... ... ... ... 198

**Closed Ward Reopened at Norwich** ... ... 198

**The Institutional Library** .. ... ... ... 199

Feeble-Mindedness in Children of School Age—A Guide to Anatomy (for Students of Medical Gymnastics, Massage, and Medical Electricity)—Gout—The Art of High Health—The Assessment of Physical Fitness.

**The Editor's Letter-Box** ... .. ... ... 200

The Registration of Scottish Fever Nurses—Dangerous Drugs Act—Discharging Patients for Lack of Nurses.

**The Matrons' and Sisters' Department** ... ... 201

A Temple of Harmony.

Some Results of Nurses' Insurance.

**Round the Hospitals** ... ... ... ... 201

**The Nursing Register of England and Wales** ... 205

Opening of the Official Home.

**The College of Nursing (Limited by Guarantee)** 206

